# Contextual equivalence for accurate comparison of wearables requires transparency

**DOI:** 10.14814/phy2.70706

**Published:** 2025-12-15

**Authors:** Michael B. Dial, Margaret E. Hollander, Emaly A. Vatne, Angela M. Emerson, Nathan A. Edwards, Joshua A. Hagen

**Affiliations:** ^1^ Human Performance Collaborative, Office of Research The Ohio State University Columbus Ohio USA; ^2^ STRONG Lab, 711th Human Performance Wing, Air Force Research Laboratory Wright‐Patterson Air Force Base Dayton Ohio USA

The authors thank the team from WHOOP for their interest and engagement with our paper. We agree that as wearable technology becomes near ubiquitous in recovery and sleep monitoring, high‐quality and transparent validation research is needed both from within the companies producing sleep‐monitoring technologies and independent research groups that are not financially or contractually tied to the companies themselves. It is important to note that the philosophy of this line of validation research from the authors is to assess the user‐facing metrics available to the general consumer that would receive as a paying customer, not by requesting special or additional access to data which would only be a proxy validation to a value shown in a smartphone app. For example, validating the interbeat interval (IBI) raw data from a device does not directly validate the heart rate variability (HRV) measure shown in the smartphone app, as there are many steps in between with data processing and calculations that take place even when methods are public. Without explicit manufacturer transparency, end users–or independent researchers–cannot discern how metrics are calculated or weighted. The authors encourage vendors to take the detailed and transparent approach of companies such as Garmin and Polar, which execute and make available scientific white papers for most metrics available to the end user (Firstbeat Technologies Ltd., 2025; Polar Electro Oy, 2025). In comparison, the authors found varying, and often non‐specific, definitions of HRV across WHOOP's publicly available information, such as: “HRV is measured overnight during the deepest period of sleep” (Heart Rate Variability (HRV) Insights & WHOOP Metrics, 2025a), and “WHOOP calculates HRV using a weighted average across your entire night of sleep, with more weight given to slow‐wave sleep (SWS) and later stages of the night” (WHOOP Recovery, 2025b).

In the absence of publicly available algorithmic details of HRV calculation, in our study we compared the resting heart rate (RHR) and HRV available in the respective apps to an overnight average. The authors would like to note they historically performed extensive in‐house validation testing with WHOOP devices, where the “last slow wave sleep phase” was manually parsed (as labeled by WHOOP timestamps in the WHOOP hypnograms) and processed in Kubios to attempt to account for this black box description. This effort showed that the accuracy of the time‐parsed data was considerably lower than an all‐night average value when compared to WHOOP's reported values. Thus, comparing the all‐night average HRV to the gold standard (instead of manually parsing sleep stage classifications from hypnograms) may have been a compromise in favor of WHOOP.

True sleep staging requires polysomnography (PSG), which directly measures the neural and physiological correlates of sleep via methods such as electroencephalography (EEG), electrooculography (EOG), and electromyography (EMG). Most wrist‐ or finger‐worn devices, however, infer sleep stages indirectly from movement and cardiovascular signals. A recent systematic review (not by our research groups) of three consumer wrist‐based devices (including WHOOP) found that the mean difference in total sleep time for WHOOP compared to PSG was −1.4 min, demonstrating excellent accuracy for sleep duration (Schyvens et al., 2024). The WHOOP also outperformed the other devices (Fitbit Charge 4 and Garmin Vivosmart 4) for multistage sleep tracking, but agreement was still only 62% accurate at identifying sleep stages (Schyvens et al., 2024). Recent meta‐analyses indicate that while wearables reliably detect sleep versus awake very accurately (sensitivities frequently >90%), performance for multistage sleep classification is much lower, with four‐stage sleep stage classification accuracies falling in the 60%–75% range (Kainec et al., 2024; Lee et al., 2025). These findings suggest that wearables are not currently sensitive enough to classify multi‐stage sleep compared to the gold‐standard methods (PSG), and thus metrics that are algorithmically weighted depending on the detected sleep stage may only introduce unnecessary error.

It should be highlighted that the WHOOP 4.0 device graded very well across the various comparisons to the ECG‐derived gold‐standard metrics in the referenced study (Dial et al., 2025). For RHR and HRV, the WHOOP 4.0 exhibited moderate levels of agreement (RHR: CCC = 0.91; MAPE = 3.00%) (HRV: CCC = 0.94; MAPE = 8.17%), strong correlations (RHR: R = 0.95) (HRV: R = 0.96), and very low levels of absolute (RHR: −1.41 ± 1.69 bpm) (HRV: −0.78 ± 5.98 ms) and relative bias (RHR: 1.78 ± 1.31 bpm) (HRV: 4.17 ± 4.33 ms) (Dial et al., 2025). Even when proprietary algorithms were treated as a “black box” and reasonable assumptions were made as to likely metric calculations, the WHOOP device was accurate, underscoring the quality of the device's hardware and software. The authors note that the differences between WHOOP and the gold‐standard in the present study fall within normal day‐to‐day variability of RHR and HRV. Normal individual variability for RHR was ~3 bpm in a longitudinal cohort of over 90,000 individuals (Quer et al., 2020). Another 16‐week study (utilizing the WHOOP 3.0) found that day‐to‐day variability in HR and HRV were ~7.5% and ~5.5%, respectively (Bellenger et al., 2022).

We appreciate WHOOP's engagement and shared commitment to advancing the ongoing validation of wearable technologies. To that end, achieving true contextual equivalence requires greater transparency from manufacturers, including public documentation of how key physiological metrics are derived. Publishing methodological briefs or white papers that outline sampling frequencies, analysis windows, and justification and processes for sleep stage weighting procedures would enable independent replication and ultimately foster trust and confidence between manufacturers, consumers, and researchers.

## AUTHOR CONTRIBUTIONS

Michael B. Dial, Margaret E. Hollander, Emaly A. Vatne, Angela M. Emerson, Nathan A. Edwards, and Joshua A. Hagen all contributed to the writing, editing, and reviewing of this letter to the editor.

## CONFLICT OF INTEREST STATEMENT

The authors declare that they have no competing interests.
